# Supplementary material for: Chromothripsis during telomere crisis is independent of NHEJ, and consistent with a replicative origin
Source: Genome Res. 2019 May;29(5):737–49. doi: 10.1101/gr.240705.118 (PMC6499312; doi:10.1101/gr.240705.118)
Supplement: Supplemental Material [file supp_gr.240705.118_Supplemental_file_1.zip › contigs/annotated_contigs/DB48/contig.3.DB48_length_918_mean_cov_31.3224400871.docx]

**DB48_length_918_mean_cov_31.3224400871**

```
C|TCTTAGGGCTTCATAAAATTAATCCATCATGTTCCTATAGCCTATTTCCTAGTGTTATCAGGTTATCTGTAAAAGCAATTCCCTTAC
  >chr7:49466425-49466839 - E=4e-234
AGATATTACTGAGAGACTTGACCTTGAATTCCCTAAGAGAAGACAGGGCTATACTTGGACATTATTGTCTGAAGCTAATGTATTCATTA

AAATAAGCTGGAGCCATTCGGTCAGCTCTCCTTTTTCCTGGGCTTCCCCATCCTCTTTATTCACAGTCGCATTCTGGACTCCATGTTAG

CTCGTGATCATTCAAGTTCTCATCTTTGGTGCGTGTATCTGAGTCTACTTTAATCTCTCAGCTCTCCTAACTTTCTCCCACCTAATACC

TCACCAGCATTATGTTATTTCTCTCTTCAAAATCACTCCAAAACCATTTTTTGCATTTAT|AACCTGATAACACTAGG|AAATTAGACA
                                                                              >chr7:494
GACATGATAAATGGAATCCGAAAATAAAAGGAACAGCAAACTACCACATAAAATTTTCATTTTACTAAGTATAGGTTTCCTATAGTCTG
72518-49472925 + E=7e-233
ACTTTAAGTCTTTTATTCTTCATAACTGCCAGCTTTTACACTTGGAGAAACAAGAAACACATTTCACCTAGGAAATGTTTTTTAATGCC

GAATACTGATTGGCAGAGCACAGTGAAATGACTCTCATGGCATCTGCATGTTTCTAGTCTGCTTCTACAGGGGCTCCTTAGATTTCTCT

CTGAGTATAGATGAAGTTTTCTAATTAATTTCCCTTTCTCCATCATTGCCCCGTCAGAATCTACCATAAATGTTATCCCATGATTAGCA

TATGTGTCACTTTGTTGATTTACACACACACACATACATAC|C|ATGGAATACTACTCACCCATAAAAAAGAATGAAAAATGACATTTG
                                           >chr9:197657-197711 - E=1e-02
CAAAAACAT|AGGAAAAACTAGAGAAAATTATTA
```
